# Case Report: Misdiagnosis of Maturity-Onset Diabetes of the Young as type 1, type 2 or gestational diabetes: insights from a Latin American tertiary center

**DOI:** 10.3389/fmed.2025.1613877

**Published:** 2025-09-12

**Authors:** Rossana Ruiz-Urbaez, David Males-Maldonado, Mariela Viviana Villagómez-Estrada, Carlos Reyes-Silva, Jorge Salazar-Vega, Gabriela Jaramillo-Koupermann, Diana Sosa-Copa, Enrique Gea-Izquierdo

**Affiliations:** ^1^Unit of Endocrinology and Diabetes, Eugenio Espejo Hospital, Quito, Ecuador; ^2^Unit of Endocrinology, Dr. José Eleuterio González Hospital, Monterrey, Mexico; ^3^Unit of Genetic, Eugenio Espejo Hospital, Quito, Ecuador; ^4^Faculty of Medicine, Pontifical Catholic University of Ecuador, Quito, Ecuador; ^5^Department of Medical Specialties and Public Health, Rey Juan Carlos University, Madrid, Spain; ^6^María Zambrano Program-European Union, Rey Juan Carlos University, Madrid, Spain

**Keywords:** Maturity-Onset Diabetes of the Young (MODY), diabetes misclassification, delayed diagnostic, whole-exome sequencing (WES), therapeutic approach

## Abstract

**Background:**

Maturity-Onset Diabetes of the Young (MODY) comprises monogenic, non-syndromic forms of diabetes inherited in an autosomal dominant pattern. MODY is frequently misdiagnosed as type 1 diabetes (T1D), type 2 diabetes (T2D), or gestational diabetes mellitus (GDM). Studies suggest that 50–90% of MODY cases are erroneously classified as type 1 or type 2 diabetes, and up to 5% of women with GDM may have undiagnosed MODY. However, data regarding the clinical presentation and genetic characterization of MODY in Latin American populations remain scarce. This study aimed to describe the clinical, analytical, and genetic characteristics of MODY patients initially misdiagnosed as T1D, T2D, or GDM in a Latin American tertiary care center.

**Methods:**

Medical history, clinical and laboratory data were obtained from electronic medical records to assess diagnostic accuracy and identify phenotypic patterns suggestive of MODY. Whole exome sequencing (WES) was employed to detect mutations related to monogenic variants.

**Results:**

We identified five patients with MODY. The median age at diabetes diagnosis was 13.6 years, while the median age at MODY diagnosis was 25.8 years. The average duration between the initial diabetes diagnosis and confirmation of MODY was 12.2 years. None of the patients presented with diabetic ketoacidosis at the onset of diabetes. All patients tested negative for islet cell autoimmunity. Of the five patients, two were initially misclassified as having T1D, two as T2D, and one as GDM. Whole-exome sequencing (WES) identified a pathogenic missense variant, c.94G>A (p.Gly32Ser), in the *INS* gene (MODY10) in one patient initially diagnosed with T1D. Another patient, also misclassified as T1D, carried a pathogenic missense variant, c.709A>G (p.Asn237Asp), in the *HNF1A* gene (MODY3). Additionally, two patients initially diagnosed as T2D were found to carry missense variants: a likely pathogenic variant, c.613G>T (p.Asp205Tyr) in the *GCK* gene (MODY2) and pathogenic variant, c.4135C>T (p.Arg1379Cys) in the *ABCC8* gene (MODY12), respectively. The patient initially diagnosed with GDM was revealed to have a pathogenic frameshift variant, c.616dupC (p.His206Profs^*^38), in the *NEUROD1* gene (MODY6). Based on these findings, a change in therapeutic approach was implemented.

**Conclusions:**

MODY is often misdiagnosed, leading to delays in appropriate management. Whole-exome sequencing is crucial for identifying pathogenic variants, enabling accurate reclassification and tailored therapy.

## Introduction

Diabetes Mellitus (DM) is an increasingly prevalent and heterogeneous chronic disease comprising clusters of phenotypically distinct forms (1). It is conventionally classified into three primary categories. These include type 1 diabetes (T1D), resulting from autoimmune β-cell destruction, usually leading to absolute insulin deficiency; type 2 diabetes (T2D), characterized by insulin resistance and a progressive decline in insulin secretion; and gestational diabetes mellitus (GDM), diagnosed in the second or third trimester of pregnancy in women without overt diabetes prior to gestation (2).

However, in the spectrum of DM, there exist atypical forms that do not align neatly with the traditional classifications of type 1 or type 2 diabetes (1). Monogenic diabetes is a rare form of diabetes caused by a single genetic mutation in one of over 40 identified genes (2, 3). The main subtypes include Maturity-Onset Diabetes of the Young (MODY), neonatal diabetes mellitus (NDM), and syndromic diabetes (3, 4). Since its initial discovery in 1974 by Fajans and Tattersall (5, 6), MODY has been recognized as the most prevalent form of monogenic diabetes, characterized by early onset of DM—typically before 25 years of age—an autosomal dominant inheritance pattern, absence of pancreatic β-cell autoimmunity, and preserved C-peptide levels (7).

MODY accounts for approximately 0.5–5% of all diabetes diagnoses and around 3.5% of those occurring before the age of 30 (3, 7). Its global prevalence varies widely across regions due to differences in clinical criteria and diagnostic practices (8–12). In Caucasian populations, mutations in the *GCK, HNF1A*, and *HNF4A* genes are responsible for 80–90% of genetically confirmed diagnoses (13, 14). In contrast, a recent study conducted in India found *HNF1A* mutations to be the most prevalent, accounting for 32.5% of MODY cases (11). However, their frequency is significantly lower in Asian populations, suggesting underlying ethnic and genetic variability in the expression of monogenic diabetes (15–17).

Misclassification of MODY is common due to its overlapping clinical features with both type 1 diabetes (T1D)—such as early onset and low body mass index—and type 2 diabetes (T2D), including preserved β-cell function and a positive family history. The broad clinical spectrum of MODY, combined with the absence of clear genotype–phenotype correlations—particularly in rare or low-penetrance subtypes—further complicates accurate diagnosis (18, 19). Consequently, an estimated 50–90% of MODY cases are incorrectly diagnosed as T1D or T2D (15), and up to 5% of women diagnosed with gestational diabetes mellitus (GDM) may, in fact, have undetected MODY (20). Despite its significant clinical implications, this frequent misclassification leads to delayed diagnosis, inappropriate treatment, and suboptimal disease management. This issue is particularly pressing in Latin America, where data on the clinical and genetic characterization of MODY are limited.

In this study, we present the clinical, biochemical, and genetic profiles of patients with MODY who were initially misdiagnosed as having T1D, T2D, or GDM in a Latin American tertiary care center. We also highlight the value of whole-exome sequencing (WES) as a tool for improving diagnostic accuracy and guiding personalized therapy in monogenic diabetes.

## Methods

Medical history, clinical and laboratory data were obtained from electronic medical records to assess diagnostic accuracy and identify phenotypic patterns suggestive of MODY. The whole exome sequencing (WES) was used to detect mutations related to monogenic variants.

## Results

Clinical and genetic characteristics of the cases in Table 1.

**Table 1:** Clinical and genetic characteristics of MODY cases (*n* = 5).

**Characteristics**		**Case 1**	**Case 2**	**Case 3**	**Case 4**	**Case 5**
Sex		Male	Female	Female	Male	Male
Family history of diabetes		Mother and grandmother	Father and grandmother	Mother, aunts, cousins, and grandmother	Mother, brother, grandfather	Father
Age of clinical diagnosis (years)		9	19	19	13	8
Ketoacidosis		No	No	No	No	No
Initial diagnosis		T1D	T2D	GDM	T2D	T1D
Exeter Mody calculator		8.20%	12.60%	12.60%	75.50%	Cannot be calculated
Age at the diagnostic of Mody (years)		22	27	45	18	17
Years of diagnostic delay		13	8	26	5	9
At diagnosis	Fasting glucose	91.9 mg/dL	93 mg/dL	107 mg/dL	130 mg/dL	472 mg/dL
	Fasting C-peptide	0.78 ng/mL	0.07 ng/mL	0.18 ng/mL	1 ng/mL	0.64 ng/mL
	HbA1c	6.6%	7.97%	6.85%	6.28%	18.66%
	CK-EPI	119 ml/min/1.73 m^2^	131.71 ml/min/1.73 m^2^	113.59 ml/min/1.73 m^2^	127.67 ml/min/1.73 m^2^	188 ml/min/1.73 m^2^
	Proteinuria	106.79 mg/24 h	Test was not taken	60.68 mg/24 h	77.28 mg/24 h	2,902 mg/24 h
	Autoantibodies (GAD65, IA2)	Negative	Negative	Negative	Negative	Negative
	Initial therapy	Insulin 0.5 IU/kg/day	Insulin 0.42 IU/kg/day	Insulin 0.73 IU/kg/day	None	Insulin
Genotype	Gen	*HNF1A*	*ABCC8*	*NEUROD1*	*GCK*	*INS*
	Nucleotide change	c.709A>G	c.4135C>T	c.616dupC	c.613G>T	c.94G>A
	Aminoacid	p.Asn237Asp	p.Arg1379Cys	p.His206Profs^*^38	p.Asp205Tyr	p.Gly32Ser
	Acmg Classification	Pathogenic	Pathogenic	Pathogenic	Likely pathogenic	Pathogenic
	Segregation	No tested	Son	No tested	Mother, brother, grandfather	*De novo* mutation
At last follow-up	Fasting glucose	76 mg/dL	110 mg/dL	176 mg/dL	110 mg/dL	114 mg/dL
	HbA1c	6.78%	6%	8.46%	6.77%	8.63%
	CK-EPI	121.6 ml/min/1.73 m^2^	126.89 ml/min/1.73 m^2^	109.67 ml/min/1.73 m^2^	104.58 ml/min/1.73 m^2^	88.02 ml/min/1.73 m^2^
	Current therapy	Glibenclamide 5 mg once daily + Insulin glargine + lixisenatide 16 IU/8 μg once daily	Glimepiride 4 mg daily	No change	None	Insulin glargine + regular insulin 1 IU/kg/day + empagliflozin 25 mg daily

### Case 1

A 23-year-old male South-American Ecuadorian patient attended a consultation in 2022. He had no apparent maternal-fetal complications but had a positive family history of presumed type 2 diabetes in his mother and grandmother. The patient presented with hyperglycemia of 466 mg/dL without ketoacidosis at the age of 9 years and was initially diagnosed with type 1 diabetes (T1D). On physical examination his weight at admission was 64 kg, but it gradually increased to 78.7 kg, reaching a body mass index (BMI) of 29 kg/m^2^. He was treated with a low-dose insulin regimen, consisting of basal insulin glargine at bedtime and prandial insulin glulisine, titrated to a total daily dose (TDD) of 0.5 IU/kg/day. This regimen achieved optimal metabolic control, with hemoglobin A1c of 6.6%. However, several factors raised doubts about the T1D diagnosis. In addition to his relatively low insulin requirements and absence of ketoacidosis, his fasting C-peptide levels remained at the lower limit of normal at 0.78 ng/mL even after 13 years of disease progression. Furthermore, his glutamic acid decarboxylase (GAD) and tyrosine phosphatase islet antigen-2 autoantibodies were negative. Although the patient's MODY probability calculator score was 8.2%, genetic panel sequencing for monogenic diabetes was performed. The results identified a pathogenic missense variant, c.709A>G (p.Asn237Asp), in the *HNF1A* gene (NM_000545.8). Following the discovery of this mutation, he was successfully transitioned from a basal-bolus insulin regimen to a combination therapy with glibenclamide (5 mg once daily), and insulin glargine/lixisenatide (iGlarLixi) (16 IU/8 μg once daily), achieving a similar hemoglobin A1c of 6.78%, with a weight reduction of 5 kg in 2 months and the omission of premeal boluses.

### Case 2

A 27-year-old female South-American Ecuadorian presented to our diabetes clinic with a history of type 2 diabetes, first diagnosed at age 19, with a blood glucose level of 350 mg/dL, hypertriglyceridemia of 1,000 mg/dL (0–150 mg/dL), and no ketoacidosis. Her family history was notable for diabetes in her father and paternal grandmother. Additionally, 2 years ago, she had a child who was diagnosed with diabetes at 2 months of age, following a pregnancy complicated by gestational hypertension. She was initially treated with glibenclamide/metformin (2.5/500 mg) before breakfast. Due to inadequate metabolic control, her regimen was switched after 3 months to NPH insulin (16 IU sc at 7 am), and regular insulin (3 IU sc at lunch). This adjustment led to episodes of hypoglycemia, prompting the discontinuation of bolos insulin and a transition to insulin glargine (24 IU sc at 9 pm). At the time of admission, her hemoglobin A1c was 7.97%, with a glucose level of 93 mg/dl, a C-peptide of 0.07 ng/mL, and negative antibodies. The estimated probability of MODY was 12.6% (≥10% for insulin-treated patients). Given her clinical profile, which was inconsistent with typical T2D, whole exome sequencing (WES) was performed, revealing a pathogenic missense variant c.4135C>T (p.Arg1379Cys) in the *ABCC8* gene (NM_000352.6).). This dominant gain-of-function variant affects the nucleotide-binding domain 2 (NBD2) of the SUR1 subunit. The overactive mutant channels lead to hyperpolarization of the beta cell membrane, reduced calcium influx through voltage-gated calcium channels, and impaired insulin secretion, which also results in low C-peptide levels due to its co-secretion with insulin. Based in this finding, glimepiride was titrated up to 4 mg daily with a progressive reduction of insulin glargine until it was discontinued. The patient subsequently experienced normalization of blood glucose and hemoglobin A1c levels.

### Case 3

The third case was a 45-year-old female South-American Ecuadorian who was diagnosed with gestational diabetes during her first pregnancy at 12 weeks of gestation, at 19 years old. At the time of diagnosis, her BMI was 20 kg/m^2^, and she did not experience ketoacidosis. However, her hyperglycemia persisted after postpartum, indicating the development of permanent diabetes. Upon admission to the clinic, the patient was being treated with glargine and regular insulin, achieving optimal metabolic control with fasting blood glucose levels of 107 mg/dL and hemoglobin A1c of 6.85%. Her C-peptide was markedly reduced at 0.18 ng/mL. Testing for diabetes-associated antibodies was negative. She reported a strong family history of apparent type 2 diabetes, affecting her mother, aunts, cousins, and grandmother. Her MODY probability calculator score was 12.6%. Genetic analysis confirmed the presence of a pathogenic frameshift variant, in the ***NEUROD1*** gene specifically c.616dupC (p.His206Profs^*^38) (NM_002500.5). No modifications were made to her treatment.

### Case 4

A 21-year- old male South-American Ecuadorian, product of a first gestation, with a perinatal history of small for gestational age (SGA) status, was diagnosed with apparent type 2 diabetes at the age of 13 years. One year later, he was additionally diagnosed with epilepsy and autism spectrum disorder. At 18 years of age, he was referred to our service for further evaluation. On physical examination, his BMI was 15.8 kg/m^2^, and relevant findings included microcephaly and right-sided hemiparesis. He had no history of diabetic ketoacidosis. Laboratory assessment revealed a C-peptide level of 1.0 ng/mL, with plasma glucose of 130 mg/dL, and islet autoantibodies were negative. The patient had never received pharmacological treatment for his diabetes. Family history included an 11-year-old brother diagnosed with diabetes. According to the Exeter MODY probability calculator, his score was 75.5%, indicating a high likelihood of monogenic diabetes. Genetic testing identified a likely pathogenic missense variant, c.613G>T (p.Asp205Tyr), in the ***GCK*** gene (NG_008847.2), which was also detected in his brother, his mother, and his paternal grandfather.

### Case 5

A 17-year-old male American Ecuadorian patient, with a paternal history of type 2 diabetes presented with hyperglycemia without ketoacidosis at 8 years, when he was diagnosed with type T1D and was started on intermediate acting insulin NPH with poor adherence. At age 17, he came to the emergency room with a 2-month history of facial and lower limb edema. Laboratory tests showed a fasting glucose of 472 mg/dL, hemoglobin A1c of 18.66%, and detectable C peptide at 0.64 ng/mL. Ketones were negative. Glutamic acid decarboxylase (GAD) and tyrosine phosphatase islet antigen-2 autoantibodies were negative. He also had glomerular hyperfiltration (CKD-EPI 188 mL/min/1.73 m2) and proteinuria close to nephrotic range (2902 mg/24 h). WES study was performed where a pathogenic missense variant, c.94G>A (p.Gly32Ser), in the ***INS*** gene. Sanger sequencing was negative in samples of the patient's first-degree relatives, assuming “*de novo*” variant in the family. The patient was treated with basal insulin glargine at bedtime, and prandial insulin regular (titrated to total daily dose 1/UI/kg/day) plus empagliflozin 25 mg orally daily. After 3 years of regular follow-up, hemoglobin A1c dropped from 18.66% to 8.6%, and proteinuria decreased from 2902 mg/24 h to 1082.65 mg/24 h (60% decrease) (21).

## Discussion

Maturity-Onset Diabetes of the Young (MODY) encompasses genetically heterogeneous, autosomal dominant forms of diabetes characterized by impaired insulin secretion due to pancreatic beta-cell dysfunction (22). Different MODY subtypes have been identified (23), with underlying pathophysiological mechanisms including: transcriptional regulation disorders involving dysfunctional nuclear transcription factors; enzyme deficiencies impacting metabolic pathways; protein misfolding disorders; ion channel dysfunctions; and signal transduction abnormalities (24, 25). These mechanistic categories result in a broad clinical spectrum ranging from relatively stable blood glucose levels to progressive deterioration in insulin secretion and extra-pancreatic features such as macrosomia, renal cysts, and azoospermia (26).

Despite the well-defined molecular underpinnings of MODY, the absence of a standardized diagnostic algorithm contributes to diagnostic delays and therapeutic mismanagement. A comprehensive diagnostic approach involves detailed clinical evaluation, diabetes-specific laboratory testing, and confirmatory genetic analysis to identify the causative mutation (25).

Given the natural history of MODY, the availability of cost-effective treatment options, and its potential impact on multiple family members, early and accurate diagnosis is essential. The presence of a positive family history, clinical features inconsistent with type 1 or type 2 diabetes, and the absence of diabetes-associated autoantibodies support the consideration of MODY in the differential diagnosis (27). However, the primary barrier to accurate diagnosis is the limited clinical recognition of monogenic diabetes among healthcare providers. Many physicians are unfamiliar with MODY and may not consider genetic testing, which is essential for a definitive diagnosis. This lack of awareness, combined with the phenotypic similarities to more common diabetes types, contributes to the high rate of misdiagnosis (28).

In our study we identified five patients with MODY. The median age at diabetes diagnosis was 13.6 years, while the median age at confirmed MODY diagnosis was 25.8 years, resulting in an average diagnostic delay of 12.2 years. This finding aligns with existing literature, which reports that the diagnosis of MODY is often delayed by more than 10 years after the initial presentation of diabetes (12).

Clinical misclassification remains a key challenge. Patients 1 (with an *HNF1A* gene mutation, MODY3) and 5 (with an-*INS* gene mutation, MODY10) were initially diagnosed with type 1 diabetes mellitus (T1D) due to the immediate requirement for insulin therapy at diagnosis. However, both lacked ketoacidosis and tested negative for pancreatic islet autoantibodies. This presentation is atypical for T1DM and raises the possibility of MODY (29). Although traditional MODY diagnostic criteria emphasize a family history of diabetes, sporadic de novo mutations—as observed in case 5—can confound this criterion. Studies have shown that de novo mutations in common MODY genes such as *HNF1A, HNF4A*, and *GCK* occur in approximately 7% of MODY cases without a family history, underscoring the importance of considering MODY even in the absence of familial diabetes (30).

Patients 2 and 4 were initially diagnosed with T2D as they did not require insulin therapy; patient 2 harboring an *ABCC8* gene mutation (MODY12) was started on metformin/glibenclamide, while patient 4, with a *GCK* gene mutation (MODY2), did not receive pharmacological treatment. Nonetheless, the absence of insulin resistance indicators—such as acanthosis nigricans, skin tags, or metabolic syndrome features—combined with negative pancreatic autoantibodies, a fasting C-peptide level exceeding 0.60 ng/mL, and a family history of early-onset, non-obese diabetes, should prompt consideration of MODY (29).

Patient 3 carrying a *NEUROD1* gene mutation (MODY6) was initially diagnosed with gestational diabetes mellitus (GDM). However, the detection of hyperglycemia before 24 weeks of gestation in a woman under 25 years old, with a normal body mass index (BMI) suggests the possibility of preexisting diabetes, such as MODY. MODY should be considered, especially when fasting glucose levels are persistently elevated ≥5.5 mmol/L (99 mg/dL) and the body mass index (BMI) is below 25 kg/m^2^. Applying these criteria, approximately 2.5% of GDM cases may warrant MODY genetic testing (20, 31). Differentiating MODY from gestational diabetes is crucial, as maternal glycemic control, which necessitates specific therapeutic approaches, and the presence or absence of mutations in the fetus can significantly impact pregnancy outcomes (32).

The MODY probability calculator, developed by the University of Exeter, has been validated as a screening tool for MODY diagnosis. A probability score of ≥20% for non-insulin-treated individuals and ≥10% for insulin-treated individuals indicate the necessity for genetic testing as per the National Health Services (NHS) England guidelines (33). Retrospective application in our cohort showed that 3 out of 5 cases exceeded these thresholds early in their clinical course, indicating that earlier testing could have expedited diagnosis.

Identifying specific subtypes of MODY is crucial for implementing precision therapy, as each subtype responds differently to treatment based on its genetic cause. For instance, the correct identification of *ABCC8*-MODY enables the switch from insulin to the use of sulfonylureas as a specific therapy, as these drugs bind to the SUR1 subunit and close the KATP channel to release insulin (34). Sulfonylurea-based therapy has been associated with improved C-peptide levels and metabolic control, and lower rates of hypoglycemia. At the same time, insulin discontinuation in these individuals favors body weight loss and reduces glucose variability, which is a potential driver for diabetic vascular complications (35).

Similarly, patients with *HNF1A*-MODY exhibit high sensitivity to sulfonylureas and may also respond to GLP-1 receptor glucagon-like peptide-1 receptor agonists (GLP-1RA). Current experience with GLP-1RA use in MODY is limited; however, these agents have demonstrated potential in reducing hypoglycemia and improving cardiovascular risk factors (26, 36).

To date, the largest MODY registry from Latin America includes 104 patients from Brazil (37). Two key studies from that country have significantly advanced the understanding of MODY genetics by identifying mutations primarily in *GCK, HNF1A*, and *HNF1B* genes (37, 38). Nevertheless, a notable limitation of these reports is the restricted exploration of rarer MODY subtypes, partly due to their focus on targeted gene sequencing. Our study contributes valuable data to the still scarce body of research on MODY in this region. There is a critical need for increased access to genetic testing, currently limited by its high cost and availability, and the integration of genetic counseling into local diabetes care protocols.

## Conclusion and recommendations

Although our case series is limited in size, it revealed both common and rare MODY subtypes, reflecting the genetic and phenotypic heterogeneity of monogenic diabetes. These findings underscore the importance of maintaining a high index of suspicion for MODY in patients with atypical diabetes presentations, regardless of the presence or absence of a family history. Accurate diagnosis, supported by WES, enabled personalized therapeutic adjustments in most cases, reinforcing the clinical value of genetic testing. Expanding access to molecular diagnostics and promoting clinician awareness are critical steps toward reducing diagnostic delays and improving outcomes. Further multicenter studies with larger cohorts are needed to validate these findings and enhance our understanding of the MODY spectrum in Latin American populations.

## Data Availability

The datasets presented in this study can be found in online repositories. The names of the repository/repositories and accession number(s) can be found in the article material.
